# Evaluation of dentists’ awareness, knowledge, and clinical practices regarding early-stage oral cancer lesions in Türkiye: A cross-sectional study

**DOI:** 10.1371/journal.pone.0341849

**Published:** 2026-02-19

**Authors:** Sayna Behkar, Paniz Golchini, Ömer Faruk Kocamaz, Serpil Altundoğan

**Affiliations:** 1 Department of Periodontology, Faculty of Dentistry, University of Health Sciences, Ankara, Turkey; 2 Department of Orthodontics, Faculty of Dentistry, University of Health Sciences, Ankara, Turkey; 3 Department of Oral and Maxillofacial Surgery, Faculty of Dentistry, Ankara University, Ankara, Turkey; King Faisal University, SAUDI ARABIA

## Abstract

This cross-sectional study aimed to evaluate dentists’ awareness, knowledge, and clinical practices regarding early-stage oral cancer lesions, with an emphasis on diagnostic behavior, patient education, and referral approaches. A total of 263 dentists from public and private institutions in Ankara, Türkiye, participated in the survey, which included a content-validated 36-item questionnaire covering demographic data, examination habits, knowledge of lesion features, biopsy practices, and educational experience. The data were analyzed via descriptive statistics, chi-square tests, and Monte Carlo simulations to explore associations between professional characteristics and clinical behaviors. While 78% of the participants acknowledged the dentist’s role in reducing oral cancer mortality, only 17.5% reported performing routine oral cancer screenings. Biopsy practice was limited, with only 11% indicating that they had ever performed a biopsy, and 36.9% preferred to monitor lesions instead of referring them immediately. Experienced dentists were significantly more likely to examine their lymph nodes (p = 0.006) and conduct routine screenings (p < 0.001). Although tobacco and alcohol use are widely recognized as risk factors, only 27.7% of the participants routinely examined high-risk anatomical areas. Patient education was reported by 92.4% of the participants, but brochures and visual aids were rarely used. Fewer than one-third of the participants rated their undergraduate training on oral cancer as sufficient, and most supported mandatory continuing education. The findings reveal considerable gaps in dentists’ preparedness and implementation of early detection strategies despite high awareness levels. Strengthening diagnostic education in undergraduate programs and promoting structured continuing education may improve early detection efforts and reduce oral cancer-related morbidity and mortality.

## Introduction

Oral cancer represents a major global public health concern; it is one of the most common cancers in the world and ranks sixth in incidence, with an estimated 389,846 new cases diagnosed in 2022.[1,2] The mortality associated with oral cancer is expected to increase significantly [3], with an estimated global death toll of more than 460,000 deaths in 2030 and a 67% increase from 2008, as claimed by the International Dental Health Foundation [3]. Although there have been several achievements in the early detection and treatment of oral cancer, the control of oral cancer is still a major issue because it is often occult and asymptomatic, especially in the early phase. Early recognition has been associated with improved survival outcomes, but many cases are undiagnosed or are diagnosed late for effective treatment [3,4].

As they examine and have regular contact with patients and access the oral cavity, dentists and dental professionals are well positioned to contribute to the identification of signs and symptoms suggestive of oral cancer [5,6]. Their knowledge, experience, and attitudes toward suspicious lesions may play a role in early detection and referral [5]. It is also important to understand the frequency of detailed oral examinations by dentists, the management of lesions suspected to be oral cancer, the response to early-stage lesions, the choice of diagnostic pathway and the referral cascade of specialists and the utilization of their clinical judgment to decide on follow-up [7,8]. Moreover, examining knowledge about risk indicators, perceived barriers to early identification and readiness for education are equally beneficial in demonstrating the usefulness of current education for future professional development [9,10]. Routine examination practices, recognition of suspicious lesions and preferred diagnostic and referral pathways are important components of early detection strategies [11]. Additionally, the frequency with which dentists encounter oral lesions, their perceptions of malignancy risk on the basis of lesion characteristics, and their comfort with performing biopsies or referring patients for further evaluation contribute to the diagnostic process [9,12]. Understanding the common sites and appearances of high-risk lesions—such as leukoplakia, erythroplakia, or submucous fibrosis—is also essential [5].

Additionally, dentists’ methods of patient education and delivery of advice could be a significant contributor to informing people about oral cancer. Discussion of areas of active intervention, including smoking, alcohol and poor oral hygiene, may prompt an earlier presentation of patients with symptoms of OSCC, and patients may become more compliant with routine screening [13]. Dental professionals should routinely question subjects regarding lifestyle habits and screen individuals at high risk with special attention to the anatomical areas frequently involved in early malignancies (lateral border of the tongue, floor of the mouth, and soft palate mucosa).

Education and training are also key factors for increasing early detection rates. A significant proportion of dentists claim that the undergraduate education they have received does not prepare them adequately to detect early carcinoma and handle precancerous lesions. Thus, postgraduate training in terms of seminars, congresses, internet-based learning and self-study is needed to ensure competence in patient care. The reactions of dental professionals to further education and their opinions on the question of whether it should be compulsory are important parameters for the preparedness of the profession to enhance its effects. Obstacles such as misdiagnosis of benign nodules, inadequate clinical exposure, and a scarcity of investigative tools may need to be addressed via comprehensive initiatives. For example, involving case-based teaching, simulated diagnostic exercises, and interdisciplinary training could help improve diagnostic skills. Greater availability of patient education materials (brochures, videos, and written information) can also assist in improving patient cooperation and knowledge.

Recalls and means for monitoring lesions (such as review appointments, recall systems or personal contact) are indicators of the dedication of a dentist’s approach to potentially malignant conditions. Moreover, investigating dentists’ perceptions of the contribution of oral cancer to total cancer-related mortality, as well as their perceived effectiveness in reducing the burden, may provide useful information concerning how dentists consider themselves to be responsible in relation to public health issues.

Ultimately, the success of early detection of oral cancer is determined not only by the dentist’s ability to control oral cancer but also by his or her sense of responsibility for risk factor identification, patient education and collaboration with various disciplines. Knowledge of the current awareness level, perceived barriers, and education needs of Turkish dentists may help to create a background for policy making, revision of curricula, and future research to minimize the burden of oral cancer deaths. These endeavors are essential in upskilling dental practitioners to participate more proactively in fighting against a major global health challenge.

The present study was therefore conducted to assess Turkish dentists’ awareness, knowledge, and practices through a comprehensive survey that can contribute to strengths and areas needing improvement in this area. This study aims to evaluate not only the knowledge levels of dentists in Turkey regarding oral cancer but also their application practices, patient education processes and patient referral approaches. Within the scope of the research, the types of practices dentists perform during oral cancer screening, diagnosis and treatment; the difficulties they encounter during these processes; and the educational materials they use will be examined.

## Materials and methods

The present cross-sectional study was designed to assess the perception and practice patterns of dentists in Ankara regarding the detection of oral cancer lesions. The study was approved by the Ethics Committee of Ankara University, Faculty of Dentistry (Decision Date: 10/03/2025, Decision No: 7/2). Participants were prospectively recruited between 15 March 2025 and 15 July 2025. Written informed consent was obtained from all participants prior to data collection, and the consent procedure was reviewed and approved by the ethics committee. In addition, permission was received from five state universities in Ankara and 10 private sector dental clinics. Among the 300 dentists approached, 263 completed the questionnaire, yielding a response rate of 88.3%. A total of 265 dentists working in various institutions across Ankara, Türkiye, participated voluntarily, and all the data were recorded anonymously after excluding those with missing or incomplete data. Data were gathered by directly administering the questionnaire in person.

**Fig 1:**
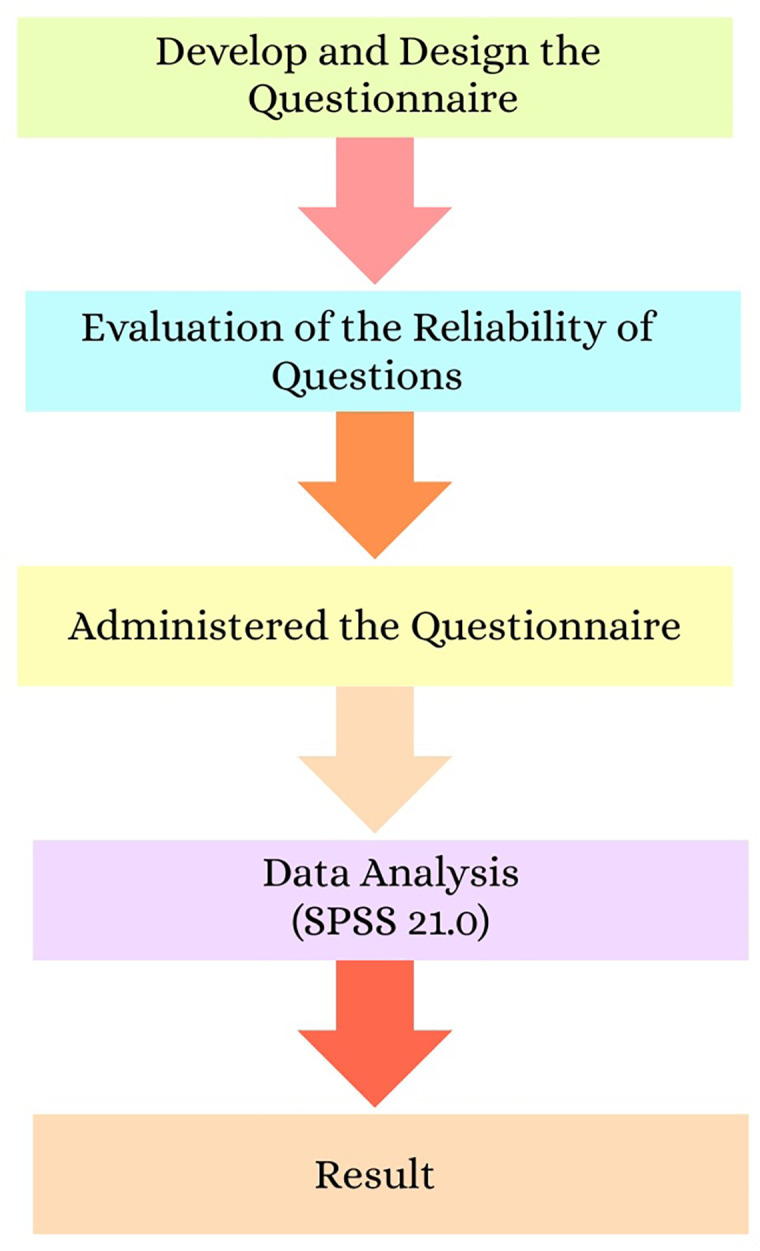
Flowchart outlining the stages of the questionnaire-based research process.

To systematically evaluate dentists’ awareness, knowledge, and practices regarding early-stage oral cancer, the process of questionnaire development, administration, and data analysis used in this study is summarized in Fig 1.

Dentists who were actively practicing in private clinics, public hospitals, or university hospitals in Ankara and who voluntarily agreed to participate were included in the study. Participants were excluded if they provided incomplete questionnaire responses.

A total of 36 questions were self-developed and consisted of multiple choices. The first 6 questions of the survey were prepared to collect demographic data such as age, title, and the specialty field of the participants. The remaining questions focused on clinical examination habits, referral behaviors, knowledge of lesion characteristics, diagnostic methods, biopsy practices, patient education habits, and views on continuing education regarding oral cancer. The survey questionnaire is provided as Supporting Information (S1 File).

An internal pilot test was conducted prior to the main study to determine the validity and reliability of the instrument using a sample of 20 participants who were not part of the entire study. Feedback from the pilot study was used to make minor revisions to the wording of the items and to improve the clarity of the response options. To assess content validity, opinions were obtained from 10 university professors. Each item in the questionnaire was evaluated for its relevance via the **content validity ratio** (CVR)**,** as described by Lawshe [14], and the **content validity index** (CVI) methodology, as recommended by Yurdugül [15]. The CVR for each item was calculated on the basis of whether the experts rated the item as “essential.” According to Lawshe’s table, for 10 experts, the minimum acceptable CVR value at the 0.05 significance level was 0.62. Items meeting or exceeding this threshold were considered to have acceptable content validity. Following individual CVR calculations, the content validity index (CVI) was computed as the mean of all CVRs, which is the overall content validity of the instrument. In this study, the average CVR (CVI) was 0.98, implying very high expert agreement. All the questions except for items 12, 17, and 18 had a CVR of 1.00. These three questions had a CVR of 0.80, were still above the required threshold, and were thus retained. To evaluate internal consistency, Cronbach’s alpha was calculated for the 30-item questionnaire. The overall reliability coefficient was found to be α = 0.927, which indicates excellent internal consistency. Power analysis was conducted prior to data collection to determine the minimum sample size. The calculation was performed with a one-sample t test set as follows: two-tailed test, effect size (g) = 0.1, alpha level (α) = 0.05, and desired power (1 – β) = 0.90. With these assumptions, the required sample size was 263 participants. This sample size will yield a 90% power for detecting a statistically significant effect given a small effect size. The achieved power with the final sample of 263 dentists was 0.90, which is sufficient statistical power for the purposes of the study.

### Data collection and analysis

To summarize the findings, descriptive statistics, which are expressed as frequencies and percentages, were used. Associations between categorical variables such as level of experience, institution type, and awareness and practice patterns were estimated with the chi-square test. When the expected cell frequencies were less than 5, the Monte Carlo simulation method was used for more reliable significance estimates. All the statistical analyses were conducted via IBM SPSS Statistics version 21.0, with the significance level set at p < 0.05.

## Results

### Demographic characteristics of the study population

The study population consisted predominantly of young dentists with limited professional experience, most of whom were affiliated with public universities. A substantial proportion of the participants were research assistants or doctoral students, reflecting the academic setting of the sample (Table 1).

**Table 1:** Demographic data of the participants.

Demographic Data		Number (n)	Percentage (%)
Age	20-30 years old	200	76.0
	31-40 years old	31	11.8
	41-50 years old	12	4.6
	51 years old ≤	20	7.6
	Total	263	100.0
Gender	Female	160	60.8
	Male	103	39.2
	Total	263	100.0
Working Sector	Private Sector	75	28.5
	Public Hospital	8	3.0
	Public University	180	68.4
	Total	263	100.0
Working experience	0-5 years	183	69.6
	6-10 years	40	15.2
	11-15 years	12	4.6
	16 years and more	28	10.6
	Total	263	100.0
Title	General Dentist	32	12.2
	Specialist Dentist	19	7.2
	University Professor	31	11.8
	Research Assistant/PhD Student	181	68.8
	Total	263	100.0
Specialty	Orthodontics	20	7.6
	Pedodontics	21	8.0
	Endodontics	33	12.5
	Periodontology	51	19.4
	Oral and Maxillofacial Surgery	61	23.2
	Oral Diagnosis and Radiology	16	6.1
	Prosthodontics	39	14.8
	Restorative Dentistry	12	4.6
	None	10	3.8
	Total	263	100.0

### Sectioned questionnaire results

**Fig 2:**
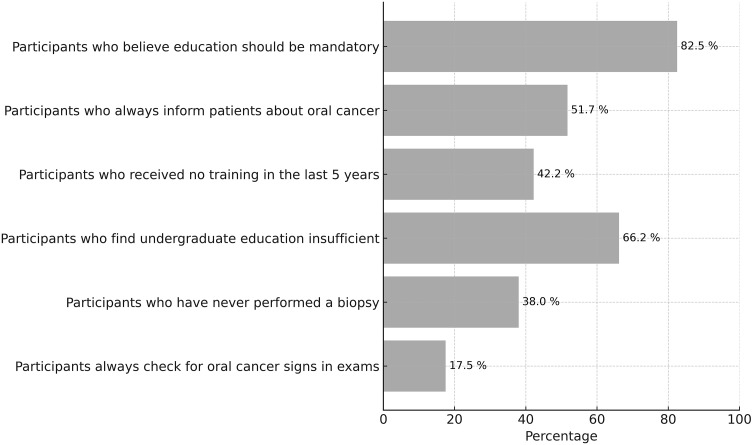
Clinical and educational status of dentists regarding oral cancer.

Dentists with more professional experience were significantly more likely to perform routine oral cancer screenings and lymph node examinations when oral lesions were detected (Table 2). These practices are less common among dentists with fewer years of experience. Routine assessment for signs of oral cancer during every clinical examination was not consistently performed among participants. Biopsy practices in the presence of suspicious lesions are limited, with many dentists reporting either infrequent or no biopsy experience. With respect to lesion monitoring, while follow-up periods consistent with the recommended timeframes were reported, prolonged observation before further diagnostic action was also commonly observed (Fig 2).

**Table 2:** Responses to Questions 1 and 15 of the survey.

		Working experience								Chi-Square Analysis	
		0-5 years		6-10 years		11 + years		Total			
		n	%	n	%	n	%	n	%	Ki-Kare	p
1. How often do you perform a detailed oral examination for signs of oral cancer during a routine check-ups?	Always	23	12.6	4	10.0	19	47.5	46	17.5	*	0.0001
	Mostly	72	39.3	16	40.0	12	30.0	100	38.0		
	Occasionally	82	44.8	20	50.0	9	22.5	111	42.2		
	Never	6	3.3	0	0.0	0	0.0	6	2.3		
	Total	183	100.0	40	100.0	40	100.0	263	100.0		
15. Do you perform a lymph node examination in the presence of oral lesions?	Always	31	16.9	15	37.5	17	42.5	63	24.0	*	0.006
	Mostly	61	33.3	12	30.0	12	30.0	85	32.3		
	Occasionally	57	31.1	8	20.0	8	20.0	73	27.8		
	Rarely	24	13.1	4	10.0	0	0.0	28	10.6		
	Never	10	5.5	1	2.5	3	7.5	14	5.3		

Upon identifying a suspicious lesion, most dentists reported referring patients to a specialist rather than initiating diagnostic procedures themselves. Referral was predominantly directed to oral and maxillofacial surgeons, with additional referrals made to otolaryngology and oncology specialists. However, a notable proportion of dentists preferred clinical monitoring before referral, indicating variability in referral practices.

**Fig 3:**
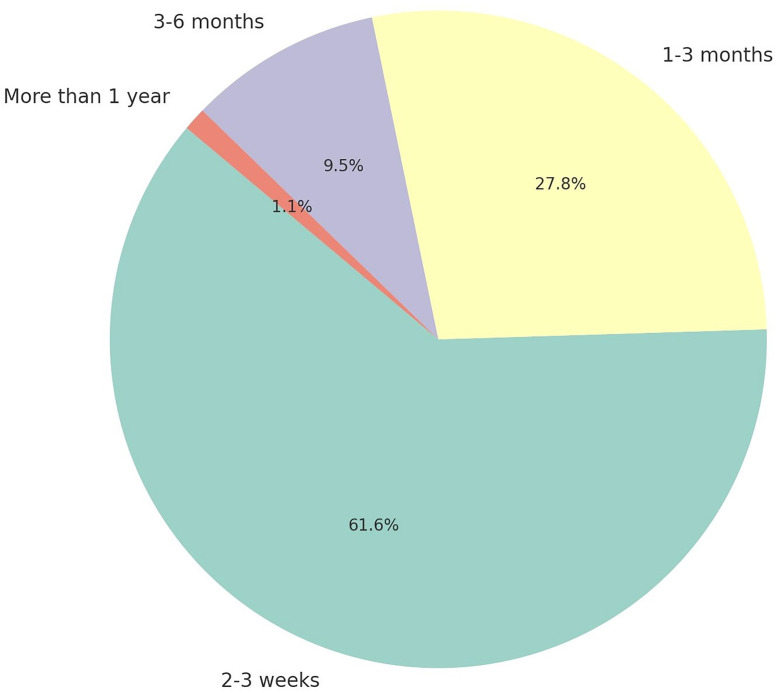
Duration of observation before suspecting malignancy in an oral lesion among participants.

Dentists most commonly reported suspecting malignancy after an initial period of clinical observation, although extended waiting periods before suspicion were also frequently noted (Fig 3). Many participants indicated that they rarely encountered oral lesions in routine practice, whereas frequent encounters were reported by a smaller subgroup. Oral lesions are predominantly observed in soft tissues, although the involvement of both soft and hard tissues is also commonly reported.

The tongue was the most frequently reported anatomical site for oral lesions, followed by the buccal mucosa and the floor of the mouth. Benign inflammatory lesions, particularly aphthous lesions, are most commonly encountered, and potentially malignant disorders such as leukoplakia and lichen planus have also been reported. Malignant neoplasms are rarely encountered. Diagnostic approaches varied among participants, with some combining biopsy with radiological and clinical examination, whereas others relied primarily on visual inspection and palpation. Irregular lesion margins are more commonly recognized as a concerning clinical feature, whereas lesion size appears to be less frequently considered.

Most participants recognized cancer survivors as being at increased risk for developing subsequent malignancies. Many dentists have reported that the clinical similarity of oral lesions poses challenges in early recognition, and a lack of education or clinical experience is also commonly identified as a barrier to accurate diagnosis. Regular examination of high-risk anatomical sites was not consistently performed, and assessment of behavioral risk factors varied among participants, with some routinely addressing these factors and others reporting doing so infrequently.

Most dentists reported preferring combined treatment approaches for oral cancer management. Among single treatment modalities, surgical intervention was selected more frequently than nonsurgical options, whereas chemotherapy and radiotherapy were reported less often.

When asked about the availability of a specialist referral network, only a minority of dentists reported having an established referral system, while many indicated that referrals were made infrequently or not at all.

With respect to patient follow-up, dentists vary in their approach to recommending regular dental check-ups for early oral cancer detection, with some advising routine follow-up for all patients and others focusing primarily on high-risk groups. Patient education regarding oral cancer was commonly reported among participants.

Follow-up intervals are most commonly determined on the basis of the basis of the clinical characteristics of the lesion, although patient-related factors and individual preferences also influence follow-up decisions. With respect to risk assessment, dentists most frequently identified behavioral factors such as tobacco use, alcohol consumption, and poor oral hygiene as major contributors to oral cancer risk. Other factors, including systemic conditions, viral infections, environmental exposures, and demographic characteristics, were reported less frequently.

Perceptions regarding the contribution of oral cancer to overall cancer-related mortality varied among participants, with some expressing uncertainty on this issue. Nevertheless, most dentists acknowledge that they play an important role in the early detection of oral cancer.

With respect to education, many dentists reported that their undergraduate training did not adequately prepare them for the early detection of oral cancer, and a substantial proportion indicated a lack of recent formal training on this topic. Nevertheless, support for continuing education on oral cancer was commonly expressed among participants.

Patient education regarding oral cancer was most commonly delivered through verbal communication, whereas the use of visual or written educational materials such as brochures or videos was limited. Follow-up practices have relied primarily on appointment-based systems or telephone contact, with digital reminders being used infrequently. To enhance professional awareness, participants most often favor case-based sharing, followed by educational seminars and scientific meetings.

**Fig 4:**
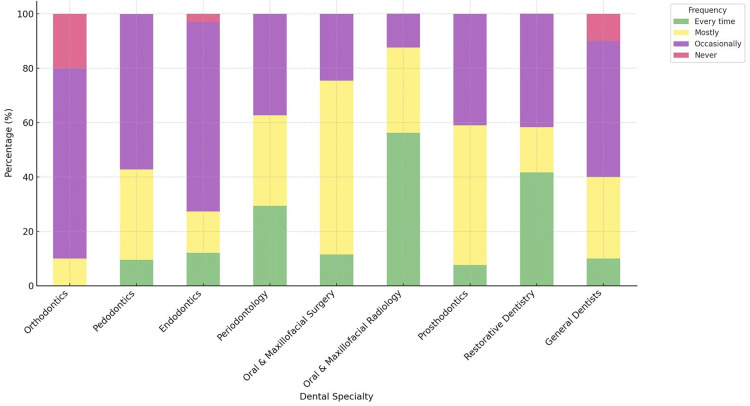
Frequency of Performing Detailed Oral Cancer Examinations During Routine Checkups by Dental Specialty.

Compliance with routine oral cancer screening varied notably across dental specialties. Oral and maxillofacial surgeons and periodontists reported higher levels of routine screening, whereas orthodontists, prosthodontists, and endodontists reported lower frequencies of regular examination. Occasional nonperformance of screening was observed across most groups, with relatively higher rates among orthodontists and nonspecialists. Fig 4 illustrates the distribution of oral cancer screening practices according to dental specialty.

No statistically significant differences were observed between professional groups regarding the perceived effectiveness of dentists in reducing oral cancer mortality (p = 0.159) or the adequacy of undergraduate training for detecting oral cancer and premalignant lesions (p = 0.215). Similarly, the frequency of checking for signs of oral cancer during routine examinations did not differ significantly by profession (p > 0.05). Lymph node examination in the presence of oral lesions showed a borderline association with the professional group, but this difference did not reach statistical significance (p = 0.098).

## Discussion

Early detection of oral cancer relies largely on systematic and routine oral mucosal examinations, placing dental professionals in a central role in timely diagnosis. [16] In the present study, comprehensive soft tissue examinations and biopsy practices were not consistently reported among participants. Limited screening behaviors and infrequent use of biopsy may contribute to delays in diagnosis, which have been associated with poorer clinical outcomes. These findings are consistent with evidence from Türkiye, where a substantial proportion of dentists reported never clinically diagnosing oral cancer despite adequate recognition of risk factors, indicating a diagnostic gap. [17] Similarly, several retrospective studies have reported low biopsy rates for premalignant and malignant lesions, supporting the possibility of underrecognition in routine clinical practice. [18–20]

On the other hand, a relatively small proportion of participants reported an active biopsy practice. This group predominantly consisted of oral and maxillofacial surgeons and periodontists, indicating that biopsy performance may vary according to professional background and clinical exposure. In line with these findings, Bhanushali et al. reported that specialists regarded biopsy procedures as critically important, whereas general practitioners more frequently reported feeling inadequately prepared to perform them. Furthermore, although most respondents reported referring suspicious lesions to oral and maxillofacial surgeons, a substantial proportion preferred clinical follow-up and re-evaluation of lesions over time. In some cases, re-evaluation intervals extended beyond commonly recommended timeframes. As described by McLeod et al., prolonged delays in diagnostic assessment may contribute to more advanced-stage diagnoses, which can adversely affect prognosis. [21]

A comprehensive meta-analysis of 17 studies involving 2,530 patients revealed that the average primary care interval (PCI)—the time between first consultation and referral—was slightly shorter for general dental practitioners (GDPs) (27.6 days) than for general medical practitioners (GPs) (30.5 days). Moreover, GDPs were more likely to refer patients at earlier stages of the disease (TNM stages I–II), indicating a potential contribution of dental practitioners to earlier detection and referral of oral cancer. [22] These findings underscore the relevance of dentists’ involvement in the diagnostic and referral process and suggest that engagement in early detection pathways may influence the stage at diagnosis.

The findings related to dentists’ perceived role in reducing oral cancer-related mortality offer important insight into their awareness of and responsibility for public health. In our study, most participants perceived dentists as having an important role in reducing oral cancer-related mortality. This finding supports findings from Horowitz et al. (2000), who reported that although U.S. dentists generally recognize oral cancer risk factors, many fail to perform comprehensive oral cancer screenings routinely, indicating a gap between awareness and clinical practice.[23] Furthermore, the World Health Organization (WHO) highlights that dentists play an important role in reducing oral cancer mortality through early detection and timely referral, which is associated with improved patient outcomes. [24]

Most participants identified aphthous ulcers (42.2%) as the most frequently encountered lesions, with only 0.4% reporting neoplasm detection, indicating that benign inflammatory lesions are commonly encountered in routine practice, whereas potentially suspicious lesions may be less frequently recognized. In Turkey, many dentists misattribute premalignant lesions to more familiar conditions, which has been associated with limited case-based and clinical exposure.[17] The findings of the present study suggest that similarities between oral lesions and limited clinical attention may complicate early detection. A lack of training and clinical exposure have also been reported as contributing factors to diagnostic difficulty. [25,26] Furthermore, most of the participants believed that their undergraduate education was inadequate or only partially adequate to provide them with competence in diagnosing oral cancer and premalignant lesions. A minority had received formal instruction on the subject in the past five years. These findings point to potential gaps in both undergraduate and continuing dental education.

In our study, 73% of the participants identified habits such as tobacco use (including cigarettes, hookah, and pipe smoking), alcohol consumption, mouth breathing, and poor oral hygiene as the major risk factors for oral cancers, which is consistent with previous research demonstrating the strong association of tobacco and alcohol use as primary risk factors [27] and the role of poor oral hygiene in increasing oral cancer risk. [28] Although direct evidence linking mouth breathing to oral cancer is limited, it has been suggested that mouth breathing contributes indirectly through mechanisms such as dry mouth and related oral health issues. [29,30] In addition, more than half of the respondents reported routinely gathering information on patients’ lifestyles and habits when assessing risk. In this context, behavioral habits are widely recognized as important factors influencing the development and progression of oral cancer.

Approximately half of the participants indicated that they rarely encountered oral lesions, which may reflect limited clinical exposure or less frequent recognition of oral lesions. A Turkish survey of dentists revealed that 67% had never been clinically diagnosed with a cancerous lesion [17], whereas a U.S. study reported that only 27.5% of general dentists frequently encounter potentially malignant lesions, with 72.5% detecting them rarely. [31] A Saudi Arabian study showed that only 30% of dentists routinely examined patients for mucosal lesions and most did so infrequently, reporting confidence and training shortage. [32] These findings indicate that limited clinical exposure or underrecognition of lesions may partly explain the low encounter rates observed in the present study. This may also be associated with variability in diagnostic routines, including lymph node examination. Awareness of the importance of lymph node examination is considered an important component of early oral cancer detection. In our study, 56.3% of the participants reported routinely assessing their lymph nodes. However, 43.7% performed this examination either limitedly or not at all. Because lymphadenopathy can be one of the most important signs of malignancy, this finding may contribute to delays in diagnosis. Notably, 42.5% of those with 11 + years of experience stated that they always perform a lymph node examination in the event of an oral lesion. These findings suggest that greater professional experience may be associated with more consistent lymph node examination practices. A study across many countries revealed that only <40% of dentists routinely palpated lymph nodes during oral cancer screening.[32] In another survey of 500 dental professionals, 54.5% of those with more than 11 years of experience examined additional-oral lymph nodes, whereas 43.4% of those with 0–5 years of experience [33].

Verbal communication is still the most common way in which dentists inform patients about oral cancer (55.9%), but the use of visual tools such as brochures and videos remains low. Although appointment-based follow-ups are frequent, more active systems such as email or SMS reminders are rarely used. Most participants viewed case sharing as the most effective way to raise awareness among dentists, indicating that practical, example-based learning may represent a valuable component of future educational approaches.

Our findings indicate notable gaps between theoretical knowledge and clinical implementation, particularly in premalignant lesion recognition and early diagnostic behavior. Similar to the recent literature, these gaps have been associated with limitations in undergraduate education, restricted clinical experience, and variability in access to continuing education. [34,35] On the other hand, this study has several limitations that should be acknowledged. First, the cross-sectional design and the use of convenience sampling restrict causal interpretations and limit the generalizability of the findings. In addition, although both specialists and general dentists were included, the predominance of university-affiliated participants may not fully reflect routine clinical practices in the broader dental community. Furthermore, reliance on self-reported data may have introduced reporting or recall bias. Therefore, the results should be interpreted with caution. Future research should involve multicenter studies with more representative samples, particularly those that include a greater proportion of general dental practitioners, as well as alternative study designs to better explore barriers to early oral cancer detection and education. Despite these limitations, the findings underscore the need for strengthened undergraduate training and structured continuing education programs to increase dentists’ competence in early oral cancer diagnosis.

## Conclusion

This study indicates that Turkish dentists’ awareness and clinical practices regarding oral cancer detection remain variable. Despite the importance of early diagnosis, many dentists do not consistently perform routine screening examinations in daily practice. These findings point to gaps in both undergraduate and continuing professional education. Therefore, encouraging training courses and providing continuous education is important to help dentists have a more active role in prevention and early detection of oral cancer. Future research should include multicenter studies with more representative samples to improve the generalizability of these findings. In addition, qualitative and interventional studies are needed to better understand barriers to early detection and to evaluate the impact of targeted educational programs.

## Supporting information

S1 File Survey questionnaire used in the study. (PDF)
