# Supplementary material for: Evaluation of dentists’ awareness, knowledge, and clinical practices regarding early-stage oral cancer lesions in Türkiye: A cross-sectional study
Source: PLoS One. 2026 Feb 19;21(2):e0341849. doi: 10.1371/journal.pone.0341849 (PMC12919796; doi:10.1371/journal.pone.0341849)
Supplement: S1 File — (PDF) [file pone.0341849.s001.pdf]

Evaluation of Turkish Dentists’ Awareness, Knowledge, and Practices Regarding Early-Stage Oral Cancer Lesions: Cross-sectional Study

Please mark the option(s) that apply to you below.

I. Your age:

☐ 20–30

☐ 31–40

☐ 41–50

☐ 51 and above

II. Your gender:

☐ Female

☐ Male

III. Sector you work in:

☐ Private sector

☐ State hospital

☐ State university

IV. Years of working experience:

☐ 0–5 years

☐ 6–10 years

☐ 11–15 years

☐ 16 years and above

V. Your professional title:

☐ Clinical dentist (general dentistry)

☐ Specialist dentist

☐ University faculty member

☐ Research assistant / PhD student

VI. Your area of specialization:

☐ Orthodontics

☐ Pedodontics

☐ Endodontics

☐ Periodontology

☐ Oral and Maxillofacial Surgery

☐ Oral Diagnosis and Radiology

☐ Prosthodontics

☐ Restorative Dentistry

☐ I have no specialization

**** Information:**

Please read the following questions carefully and mark only one option that best applies to you.

1. How often do you perform a detailed oral examination for signs of oral cancer during routine check-ups?

☐ Always

☐ Mostly

☐ Occasionally

☐ Never

2. What steps do you take when you notice early-stage symptoms of oral cancer?

☐ Refer the patient to a specialist

☐ Follow the lesion and re-evaluate if it progresses

☐ Perform an immediate biopsy

3. To which specialists do you refer patients after identifying a suspicious lesion?

☐ Oral and maxillofacial surgeons

☐ ENT (ear, nose, throat) specialists

☐ Plastic and reconstructive surgeons

☐ Oncologists

4. How long do you observe an oral lesion before suspecting malignancy?

☐ 2–3 weeks

☐ 1–3 months

☐ 3–6 months

☐ More than 1 year

5. Do you perform biopsies on suspicious lesions?

☐ Always

☐ Often

☐ Occasionally

☐ Rarely

☐ Never

6. How often have you encountered oral lesions so far?

☐ Very often

☐ Often

☐ Rarely

☐ Never

7. In which tissue types do you expect oral lesions to commonly appear?

☐ Soft tissue

☐ Bone tissue

☐ Both

8. In which regions do you expect to most commonly find soft tissue lesions in the oral cavity?

☐ Tongue

☐ Buccal mucosa

☐ Gingiva and alveolar mucosa

☐ Hard and soft palate mucosa

☐ Floor of the mouth

9. What is the most common type of lesion you encounter or suspect in oral lesions?

☐ Leukoplakia

☐ Lichen planus

☐ Erythroplakia

☐ Aphthous lesions

☐ Oral submucosal fibrosis

☐ Neoplasm

10. What methods do you use when examining lesions suspected of oral cancer?

☐ Only visual examination

☐ Visual examination and palpation

☐ Clinical exam and radiological imaging

☐ Clinical exam, radiological imaging, and biopsy

11. What criteria do you consider most when evaluating whether an oral lesion carries a risk of malignancy?

☐ Lesion size

☐ Lesion color

☐ Lesion firmness

☐ Duration of lesion

☐ Irregular lesion borders

☐ Tendency of the lesion to bleed

☐ Patient's age and gender

12. Do you think a patient with a previous diagnosis of malignancy is at higher risk for developing another?

☐ Definitely

☐ Yes

☐ Slightly

☐ Not at all

13. What do you think are the biggest challenges in the early diagnosis of oral lesions?

☐ Lack of distinctive clinical signs

☐ Confusion with similar lesions

☐ Inadequate education and experience

☐ Limited access to diagnostic tools

14. Do you examine specific areas such as the oropharynx that may show early signs of oral cancer?

☐ Always

☐ Frequently

☐ Occasionally

☐ Rarely

☐ Never

15. Do you perform lymph node examinations in the presence of oral lesions?

☐ Always

☐ Frequently

☐ Occasionally

☐ Rarely

☐ Never

16. Do you inquire about patients' lifestyle habits and risk factors for oral cancer?

☐ Always in detail

☐ Generally

☐ Occasionally

☐ Rarely

☐ Never

17. What types of treatments do you think referred patients generally receive?

☐ Surgical intervention

☐ Chemotherapy

☐ Radiotherapy

☐ Combined treatment

18. Do you have an active referral network (e.g., oral surgeons, oncologists) for prompt referrals?

☐ Yes, I frequently refer

☐ I sometimes use a referral network

☐ I rarely refer

☐ I have a network but do not use it

☐ I have no referral network

19. Do you encourage patients to have regular check-ups for early oral cancer diagnosis?

☐ Yes, I recommend it to every patient

☐ Only to patients with risk factors

☐ Rarely

☐ No, I don't inform them about it

20. How often do you think dentists should inform their patients, especially high-risk individuals, about oral cancer?

☐ Always

☐ Mostly

☐ Rarely

☐ Never

21. In the oral cancer cases you have seen, which of the following risk factors have you observed most frequently?

☐ Habits: tobacco use (cigarettes, hookah, pipe, etc.), alcohol use, mouth breathing, poor oral hygiene

☐ Environmental factors: UV radiation, malnutrition, radiation exposure

☐ Systemic diseases or genetic factors

☐ Viruses and infections (e.g., HPV, HIV, EBV)

☐ Age and gender

22. How do you determine the frequency of follow-up visits?

☐ Based on lesion status

☐ Based on risk factors

☐ Based on patient request

☐ According to routine appointment schedule

23. How significant do you believe oral cancer is among overall cancer-related deaths?

☐ Oral cancer holds a significant place

☐ Oral cancer is less influential

☐ I'm not aware of oral cancer's impact on cancer mortality

24. How effective do you believe dentists are in reducing mortality through oral cancer diagnosis?

☐ Very effective

☐ Quite effective

☐ Moderately effective

☐ Slightly effective

☐ Not effective at all

25. Do you find your undergraduate education sufficient in identifying oral cancer and premalignant lesions?

☐ Very sufficient

☐ Sufficient

☐ Somewhat sufficient

☐ Insufficient

☐ Not sufficient at all

26. Have you received any training related to oral cancer in the past five years?

☐ Yes, formal training

☐ Yes, self-taught through literature

☐ Yes, attended seminars or workshops

☐ No, but I tried to learn about it

☐ No, I have received no training

27. Do you think it would be beneficial to make continuing education programs (congresses, seminars, etc.) on oral cancer mandatory among dentists?

☐ Yes, it should be mandatory

☐ Yes, but only under certain circumstances

☐ Undecided

☐ No, it shouldn't be mandatory

☐ No, it should remain optional

28. What materials do you use to inform patients about oral cancer?

☐ Brochures

☐ Video materials

☐ Written documents

☐ Verbal communication

☐ Combination

☐ None

29. What methods do you use for follow-up?

☐ Phone calls

☐ Appointment tracking

☐ Email or message reminders

☐ Patient-initiated follow-up

30. What do you think is the most effective method to increase oral cancer awareness among dentists?

☐ Educational seminars

☐ Scientific congresses

☐ Academic publications

☐ Case sharing related to oral cancer

☐ Online courses

**Thank you for your participation. Your responses have been recorded and will be used solely for academic research purposes.
